# To Divide or Invade: A Look Behind the Scenes of the Proliferation-Invasion Interplay in the *Caenorhabditis elegans* Anchor Cell

**DOI:** 10.3389/fcell.2020.616051

**Published:** 2021-01-06

**Authors:** Evelyn Lattmann, Ting Deng, Alex Hajnal

**Affiliations:** ^1^Department of Molecular Life Sciences, University of Zurich, Zurich, Switzerland; ^2^Molecular Life Science PhD Program, University and ETH Zurich, Zurich, Switzerland

**Keywords:** anchor cell, invasion, proliferation, EGL-43, EVI1, cell cycle, basement membrane

## Abstract

Cell invasion is defined by the capability of cells to migrate across compartment boundaries established by basement membranes (BMs). The development of complex organs involves regulated cell growth and regrouping of different cell types, which are enabled by controlled cell proliferation and cell invasion. Moreover, when a malignant tumor takes control over the body, cancer cells evolve to become invasive, allowing them to spread to distant sites and form metastases. At the core of the switch between proliferation and invasion are changes in cellular morphology driven by remodeling of the cytoskeleton. Proliferative cells utilize their actomyosin network to assemble a contractile ring during cytokinesis, while invasive cells form actin-rich protrusions, called invadopodia that allow them to breach the BMs. Studies of developmental cell invasion as well as of malignant tumors revealed that cell invasion and proliferation are two mutually exclusive states. In particular, anchor cell (AC) invasion during *Caenorhabditis elegans* larval development is an excellent model to study the transition from cell proliferation to cell invasion under physiological conditions. This mini-review discusses recent insights from the *C. elegans* AC invasion model into how G1 cell-cycle arrest is coordinated with the activation of the signaling networks required for BM breaching. Many regulators of the proliferation-invasion network are conserved between *C. elegans* and mammals. Therefore, the worm may provide important clues to better understand cell invasion and metastasis formation in humans.

## Introduction

**Figure 1:**
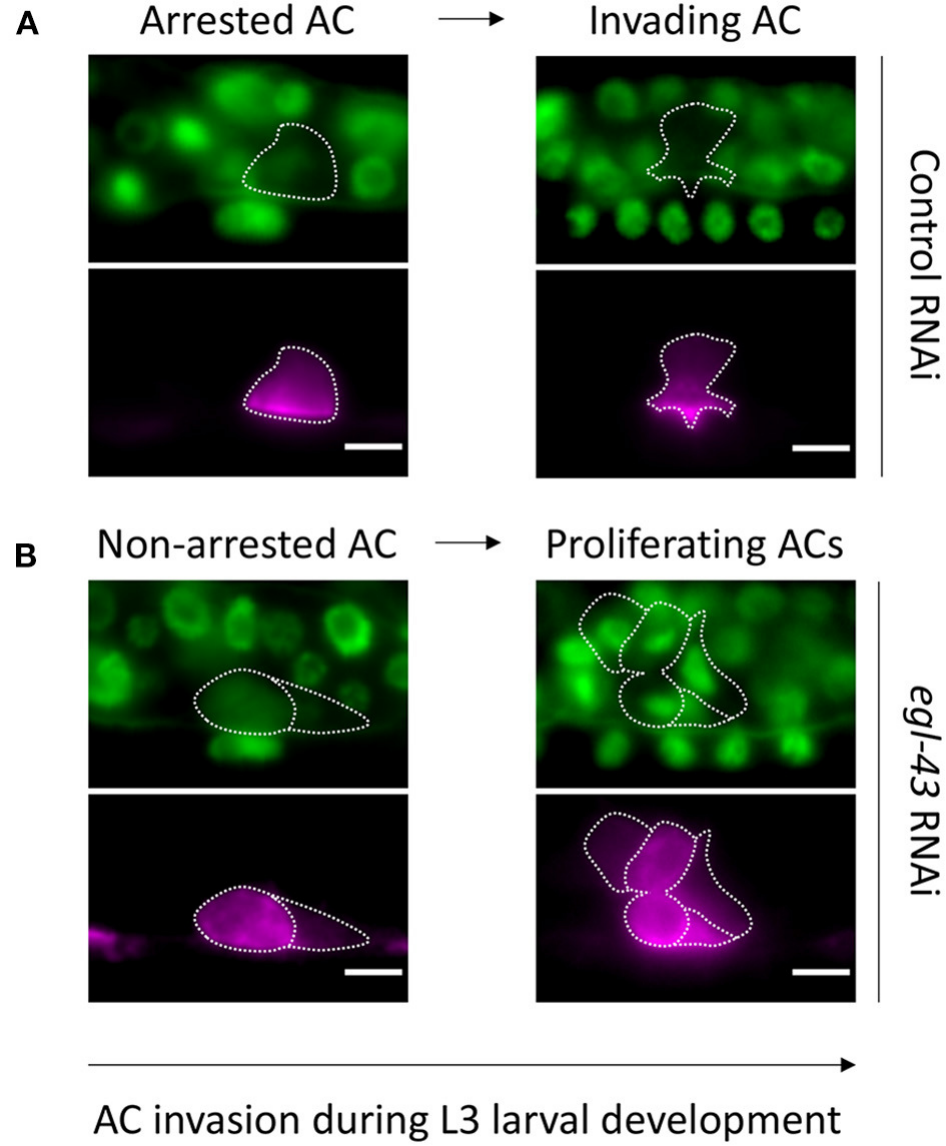
Observing AC invasion in *C. elegans* larvae: **(A)** Animals treated with control RNAi showing a non-dividing AC associated with normal invasion at the 1-cell (A′) and 4-cell (A″) stage. **(B)** The AC in *egl-43* RNAi treated animals continues to divide and is incapable of invading between the primary VPCs. Note that the left AC (B′) is in early anaphase and the middle AC (B″) is about to complete its division. **(A,B)** Upper rows depict cell nuclei with GFP::MCM-7 and the basement membrane with LAM-1::GFP. Lower rows illustrate corresponding AC as labeled with a cdh-3>mCherry::PH maker. The arrow depicts developmental timing.

AC invasion in *Caenorhabditis elegans* is an excellent model to investigate the various checkpoints regulating developmental cell invasion, including G1 cell cycle arrest required for BM breaching (Matus et al., 2015; Deng et al., 2020; Medwig-Kinney et al., 2020). AC invasion occurs during the mid- to late-L3 larval stage in order to establish a connection between the uterus and developing vulva (Sherwood and Sternberg, 2003) (Figure 1). The importance of the morphogenetic events triggered by AC invasion manifests in mutants with defective AC invasion. For example, loss-of-function mutations in the AP-1 transcription factor *fos-1 (FOS, FOSL1, FOSL2)*, the gene encoding a key invasion driver, lead to a protruding vulva (Pvl) phenotype and adult sterility (Sherwood et al., 2005). The AC is derived from one of two primordial gonadal cells, Z1 and Z4 (Kimble and Hirsh, 1979). Two of the 12 Z1 and Z4 descendant Z1.ppp and Z4.aaa, have an equal potential to adopt the default AC fate, but only one cell becomes the AC during the L2 larval stage, while the other one is acquiring the ventral uterine (VU) fate stage (Kimble and Hirsh, 1979; Kimble, 1981). A positive feedback loop established by upregulation of the Notch ligand LAG-2 (DSL) in the future AC and by lateral inhibition via activation of the Notch receptor LIN-12 (Notch) in the adjacent VU precursor underly the AC/VU decision (Seydoux and Greenwald, 1989; Greenwald and Kovall, 2013). *hlh-2* (*TCF3, TCF4,TCF12*) encodes a basic helix-loop-helix transcription factor that up-regulates *lin-12* expression in the presumptive VU cell (Attner et al., 2019). The initial imbalance in Notch signaling is driven by the onset of *hlh-2* expression, which is linked to the relative birth order of the AC/VU ancestor cells Z1.ppp and Z4.aaa. Later on, HLH-2 is post-transcriptionally silenced in the future VU cell, whereas HLH-2 in the AC binds to the E-boxes in the *lag-2* promoter, thus maintaining LAG-2 expression and establishing the positive feedback loop (Karp and Greenwald, 2004). *In vitro* assays have suggested that HLH-2 also binds to E-boxes in the promoter of *egl-43*, which encodes a zinc finger transcription factor homologous to the human EVI1 proto-oncogene and contributes to the VU/AC decision (Hwang et al., 2007; Rimann and Hajnal, 2007). While *egl-43* is important for G1 arrest and pro-invasive gene expression in the AC (see below), *egl-43* is also expressed in the proliferating VU cells where it promotes VU fate specification (Rimann and Hajnal, 2007). Moreover, HLH-2 is required in the AC to upregulate expression of the epidermal growth factor homolog LIN-3 (EGF), which acts as inductive signal during vulval cell fate specification (Hwang and Sternberg, 2004; Sternberg, 2005).

## Regulation of AC Invasion

**Figure 2:**
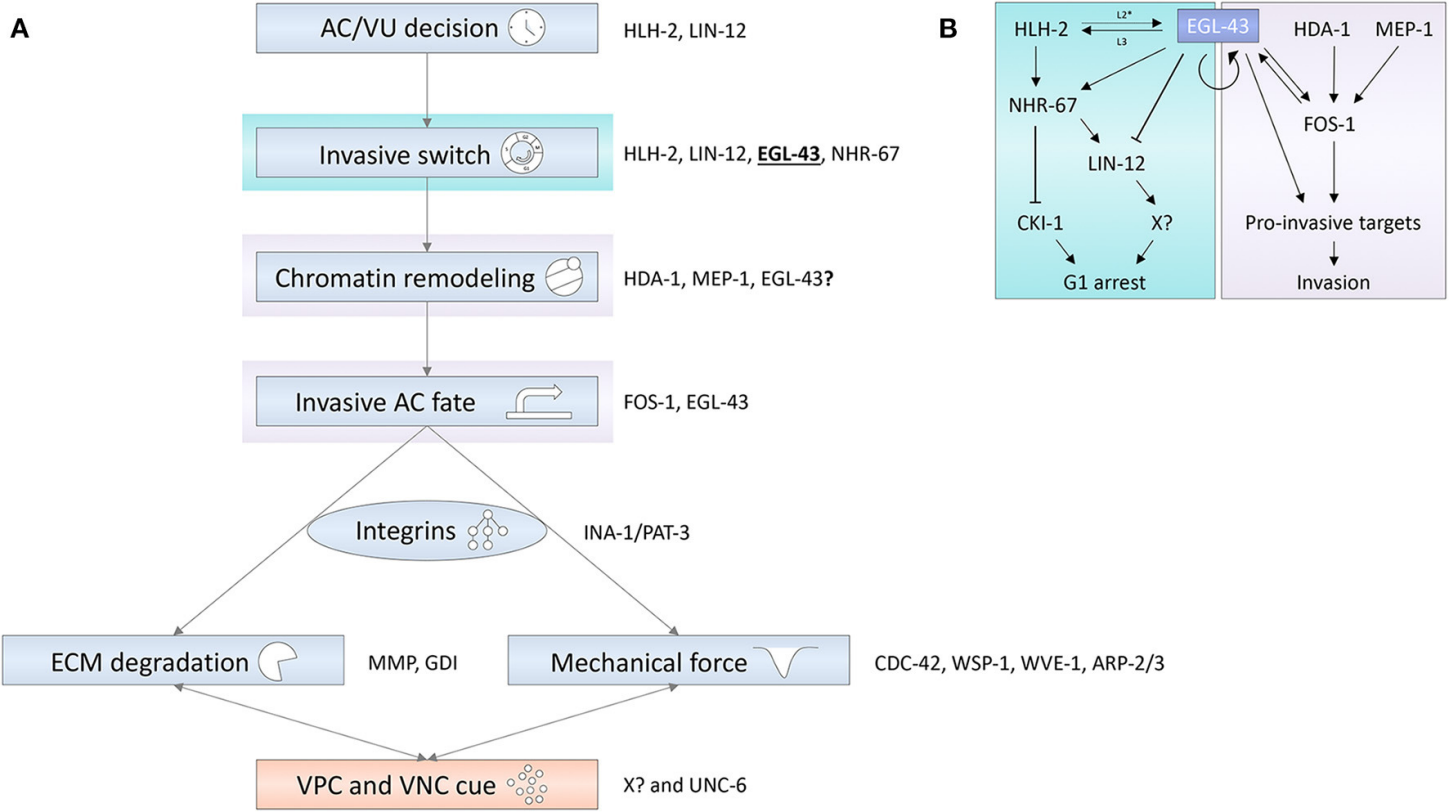
**(A)** Different regulatory layers are integrated to control AC invasion. Blue shaded boxes depict cell-autonomous processes in the AC; the red shaded box represents paracrine signaling processes mediated by the VPCs and the ventral nerve cord (VNC). Some of the genes encoding factors important for the depicted processes are shown on the right of the rectangles. **(B)** The EVI1 homolog EGL-43 is a central node in a transcription factor network coordinating the different signaling layers that induce G1 cell cycle arrest and promote pro-invasive gene expression. Arrows refer to activating and T-bars to inhibitory interactions.

While *egl-43* and *hlh-2* are important for the AC/VU fate decisions, they later play a central role in inducing the invasive AC fate by enabling G1 cell cycle arrest and activating the expression of pro-invasive genes, which are controlled by the *C. elegans* ortholog of human FOS *fos-1* (Sherwood et al., 2005) (Figure 2). Among the *fos-1* target genes are several conserved extracellular matrix genes, such as hemicentin *him-4* (*HMCN1*), the zinc metalloproteinases genes (MMPs: *zmp-1, zmp-3*, and *zmp-6*), the papilin homolog *mig-6* (*PAPLN*), or the protocadherin *cdh-3* (*PDCH*), as well as actin cytoskeleton regulators such the small GTPases *mig-2* (*RHOG*) and *ced-10* (*RAC*) or the lamellipodin homolog *mig-10* (*RAPH1*) (Sherwood et al., 2005; Ihara et al., 2011; Wang et al., 2014a; Matus et al., 2015). AP-1 also drives tumor invasion in several types of human cancer (Ozanne et al., 2006). Furthermore, there exists some degree of conservation at the level of target genes, such as MMP1, MMP3, and MMP9 that have been shown to be under direct AP-1 control in a variety of cellular contexts (Angel et al., 1987; Lee et al., 1987; Benbow and Brinckerhoff, 1997).

G1 cell cycle arrest of the AC is a pre-requisite for pro-invasive gene expression and formation of invasive protrusions, as S-phase or G2 entry of the AC prevents invasion (Matus et al., 2015). Moreover, the activity of HDA-1, a component of the nucleosome remodeling deacetylase (NuRD) complex among others, is important for linking the G1 arrest to *fos-1*-mediated gene expression and formation of invadopodia-like protrusions (Matus et al., 2010). Besides HDA-1, MEP-1 and the cytosolic chaperonin containing TCP-1 (CCT) complex act upstream of *fos-1* and actin polarity pathways. Interestingly, in the germ cells MEP-1 interacts with the NuRD complex to maintain the somatic differentiation (Unhavaithaya et al., 2002), and also the CCT complex interacts with HDAC1 (Dekker et al., 2008; Banks et al., 2018). However, how these chromatin modifiers precisely act in the invading AC remains to be studied.

A positive regulation of *fos-1* by *egl-43* has recently been observed (Deng et al., 2020; Medwig-Kinney et al., 2020). Thus, *egl-43* plays a dual role in AC invasion, on the one hand by inducing G1 cell cycle arrest and on the other hand by activating expression of *fos-1*. Despite acting downstream of the G1 arrest and *egl-43, fos-1* positively regulates *hlh-2* and *egl-43* expression in the AC, revealing a complex regulatory network formed by these three transcription factors (Medwig-Kinney et al., 2020). While *fos-1* loss-of-function mutants exhibit fully penetrant BM breaching defects, mutations in the FOS-1 target genes do not cause strong AC invasion defects (Sherwood et al., 2005). Even in *cdh-3, him-4, zmp-1* triple mutants, the AC failed to invade in only 25% of the animals (Kelley et al., 2019). These findings have suggested the existence several partially redundant invasion pathways that ensure robust BM breaching. A study in human ovarian carcinoma cells has found several overlapping targets of the AP-1 and EVI1 transcription factors (Bard-Chapeau et al., 2012). Therefore, *fos-1* targets may be co-regulated in a cooperative fashion by *egl-43. mig-10* is one example for an antagonistic regulation, whereby *fos-1* activates and e*gl-43* inhibits *mig-10* expression (Wang L. et al., 2014; Wang et al., 2014a).

Apart from the enzymatic dissolution of the BM, breaching and invasion also require physical forces generated by invasive protrusions. These forces are exerted by actin-rich protrusions that depend on the actin nucleator complex Arp2/3, which is activated in the AC predominantly by WSP-1/N-WASP and to a lesser extent by WVE-1/WAVE (Cáceres et al., 2018). Upstream of WSP-1/N-WASP acts the GTPase CDC-42 (Lohmer et al., 2016). However, MIG-2/Rac signaling can compensate for a loss of WSP-1/N-WASP activation when CDC-42 is absent (Cáceres et al., 2018). Moreover, in the absence of the entire CDC-42/Cdc-42-MIG-2/Rac-WSP-1/N-WASP pathway, WVE-1 signaling can be activated by CED-10/Rac signaling (Lohmer et al., 2016; Cáceres et al., 2018). Activation of CDC-42 in the AC depends on one or several unknown diffusible cues secreted by the induced primary vulval precursor cells, together with an UNC-6 (NTN1) Netrin signal released from the ventral nerve cord (Ziel et al., 2009; Lohmer et al., 2016). These two signals guide the AC protrusions in order to breach the BM and to invade precisely at the vulval midline between the primary VPCs (Sherwood and Sternberg, 2003). The UNC-6 Netrin signal directs the AC protrusions toward the ventral midline by binding to the UNC-40 (DCC/NEO1) receptor that is polarized toward the invasive membrane in the AC (Ziel et al., 2009). The UNC-6/UNC-40 netrin pathway is required for the polarized enrichment of actin regulators, such as phospholipid phosphatidylinositol 4,5-bisphosphate (PI(4,5)P2), MIG-2 (RHOG), CED-10 (RAC), UNC-115 (ABLIM1), and UNC-34 (EVL). Finally, UNC-40-directed lysosomal exocytosis, which delivers MMPs and membrane fractions to the invadopodia-like protrusions, leads to the formation of a single AC protrusion (Naegeli et al., 2017). However, *unc-6* and *unc-40* mutants do not exhibit identical phenotypes, since UNC-40 can function in a ligand-independent way to regulate F-actin polarity and partially compensate for the lack of the UNC-6 ligand (Wang et al., 2014b). Both *unc-40* and *unc-6* mutants display a delayed invasion, but they do not block invasion (Ziel et al., 2009), indicating that the Netrin signal and the cue from the vulval cells are partially redundant. Thus, the activation of the CDC-42 pathway and the force generation by the invasive protrusions depend on multiple cell non-autonomous cues.

In addition to the CDC-42 pathway, the vulval cue regulates the Rab GDP dissociation inhibitor (GDI), which controls membrane trafficking to form plasma membrane protrusion (Lohmer et al., 2016). The molecular nature of the signals activating the CDC-42 and GDI pathways in the AC remains an enigma. G-Protein coupled receptors (GPCR), receptor tyrosine kinases (RTKs) and integrins are known to be involved in CDC-42 activation. However, besides the Netrin cue, no other secreted signal or receptor required for AC invasion has been found to date.

Signaling through the INA-1 (ITGA3, ITGA6, ITGA7)/PAT-3 (ITGB1) alpha/beta integrin complex acts upstream of the Netrin pathway to control the recruitment of F-actin to the AC plasma membrane (Hagedorn et al., 2009). The different phenotypes of *ina-1* and *pat-3* mutants compared to Netrin pathway mutants suggest that the integrins play a broader role in recruiting UNC-40 and F-actin to the plasma membrane, while the netrin signal provides the directional information for a specific recruitment to the invasive membrane front.

## The EVI1 Homolog *egl-43* Coordinates AC Proliferation and Invasion

As discussed above, *egl-43* is necessary for G1 cell cycle arrest of the AC. In addition, the nuclear hormone receptor gene *nhr-67* (*NR2E1*) is critical for G1 cell cycle block in the AC (Matus et al., 2015). Removal of either of these two transcription factors results in AC proliferation, reduced pro-invasive gene expression, impaired invasive membrane organization and lack of invadopodia-like protrusion formation. This implies that *egl-43* and *nhr-67* are both essential for halting cell cycle progression in the invasive AC. How these transcription factors interact and coordinate the cell cycle arrest with invasion has been the subject of two recent publications.

Medwig-Kinney et al. (2020) and Deng et al. (2020) studied the relationship between *hlh-2, egl-43, fos-1*, and *nhr-67* during cell invasion and identified a network defining a cell-cycle dependent axis of invasion control, whereby *egl-43* regulates *nhr-67* expression in an *hlh-2*-dependent and -independent manner (Figure 2). NHR-67 then establishes the G1 arrest in the AC by activating expression of the CDK inhibitor CKI-1 (Matus et al., 2015). Moreover, *egl-43* positively regulates *fos-1* expression, indicating an additional, cell-cycle independent role of *egl-43* in controlling AC invasion. In this model, *egl-43* emerges as a central player linking G1 arrest to the cell-cycle independent invasion network by activating *fos-1* and *nhr-67* expression and forming several positive feedback loops including autoregulation. CHIP-seq data indicated that EGL-43 may act by directly binding to enhancer elements in the *fos-1* locus (Deng et al., 2020).

However, *egl-43* and *nhr-67* may also act in distinct pathways, since *nhr-67* establishes the cell cycle arrest in the AC primarily via CKI-1 expression, whereas *egl-43* appears to restrict AC proliferation predominantly by inhibiting the LIN-12 Notch pathway. Surprisingly, the ectopic activation of LIN-12 Notch signaling in the already differentiated AC was sufficient to induce proliferation. On the other hand, inhibiting *lin-12* notch expression efficiently suppressed the AC proliferation caused by loss of *egl-43*, but not *nhr-67* function (Deng et al., 2020). In several cell types, Notch signaling directly promotes G1-S transition. For example, Notch regulates cyclin D1 expression in mammalian kidney, breast epithelial cells and cardiomyocytes (Ronchini and Capobianco, 2001; Campa et al., 2008; Cohen et al., 2010), activates dE2F1 and cyclin A expression in the *Drosophila* photoreceptor precursors (Baonza and Freeman, 2005) and negatively regulates the CDK inhibitors p27^Kip1^ and p21^Cip1^ to promote S-phase entry (Noseda et al., 2005; Sarmento et al., 2005).

Taken together, two distinct mechanisms ensure G1 arrest of the AC; EGL-43 inhibits S-phase entry by repressing Notch signaling, while NHR-67 maintains the G1 arrest of the AC by activating CKI-1 expression. This double authentication system established by NHR-67 and EGL-43-mediated cell cycle inhibition may add the developmental robustness necessary for the AC to adopt a stable invasive fate. Further studies will be needed to probe this hypothesis and identify additional cell cycle regulators controlled by *nhr-67, egl-43* and the *lin-12* Notch pathway.

### Context-Dependent Regulation of Proliferation by *egl-43* and *lin-12* Notch Signaling

*egl-43* is not only expressed in the AC but also in the VU cells that undergo three rounds of cell division. This raises the important question of cell context-dependent specificity. In fact, the absence of terminal differentiation (π-fate) markers in the VU cells after inhibition of *egl-43* might even hint at a defect in VU cell proliferation. In the VU cells *egl-43* seems to be positively regulated by Notch signaling, which turns around the relationship observed in the AC. Whether this difference in LIN-12 activity is caused by a different type of regulation remains to be examined. DNA binding of the human EGL-43 homolog EVI1 is modulated by serine phosphorylation through casein kinase II (CK2) and PP1A (Bard-Chapeau et al., 2013). In particular these phosphorylation sites have been shown to modulate EVI-1 DNA binding to ETS-like binding motifs (Bard-Chapeau et al., 2013), often present in cell cycle regulator genes (Bard-Chapeau et al., 2012). CK2 also phosphorylates the intracellular domain of NOTCH (NICD), which results in differential binding to the Notch transcription factor complex [LAG-1 (CBF1/RBP-J)/SEL-8 (MAML)] and changes the pattern of NOTCH driven target gene expression (Ranganathan et al., 2011). Hence, differential phosphorylation of EVI1 and NOTCH may be important for their context-dependent activities. Alterations in EVI1 regulation have also been attributed to the differential expression of the long vs. the short isoform. In the *C. elegans* AC, the long isoform EGL-43L is the dominant factor regulating G1 arrest and pro-invasive gene expression, for which the short isoform is dispensable (Deng et al., 2020; Medwig-Kinney et al., 2020). One possibility is therefore that the short EGL-43S isoform plays the opposite role in promoting VU cell proliferation.

### Timing of the G1 Arrest

Besides the spatial context established through cell-cell signaling, the timing of the G1 arrest in the AC needs consideration. Studies with heterochronic mutants reveal that the timing of invasion is intrinsically programmed in the AC. For example, in a *lin-28* (*LIN28A, LIN28B*) mutant, where the primary VPC is precociously induced, the AC invades at the normal developmental time in mid-L3, but the vulva is already at the morphogenetic stage (L4 stage) (Sherwood and Sternberg, 2003). Thus, the competence to respond to extracellular invasion cues likely depends on a cell-autonomous molecular clock in the AC.

While *hlh-2, nhr-67* and *egl-43* are required for the maintenance of the G1 arrest throughout the L3 phase, the G1 arrest is already established at the L2 stage, shortly after the specification of the AC. Low *fos-1* expression can be seen in the newly specified AC and expression levels gradually increase until invasion begins (Sherwood and Sternberg, 2003; Sherwood et al., 2005; Medwig-Kinney et al., 2020). Since HLH-2, EGL-43, and NHR-67 are already expressed during the AC/VU decision, it seems likely that the induction of the G1 arrest is tied to AC fate specification at the early L2 larval stage. In this context it is important to note that the early L2 function of *egl-43* is required for AC invasion during the later L3 stage, which was shown by early expression of the dominant-negative *egl-43* PR domain in the AC (Hwang et al., 2007). An invasion defect was only observed when the PR domain was expressed from an *egl-43* promoter with functional E-boxes, required for early expression of *egl-43* at the mid L2 stage (Hwang and Sternberg, 2004; Hwang et al., 2007). Since *egl-43* expression in the newly formed AC may depend on *hlh-2* activity (Hwang et al., 2007), the onset of *hlh-2* expression could serve as a molecular clock to set the time of invasion. However, no clear timing dependency has been observed in a recent study examining at an *egl-43* reporter upon *hlh-2* RNAi at the L3 stage (Medwig-Kinney et al., 2020). Since the specification of the AC itself depends on *hlh-2*, early expression changes in the newly formed AC cannot be addressed by this approach. Different combinations of bHLH transcription factors represent distinct codes for cell fate specification (Sallee et al., 2017). Thus, *hlh-2* might integrate developmental timing with spatial cues to program the AC for the G1-arrested invasive state.

### A Pro-invasive Chromatin Landscape?

Down-regulation of *hda-1* suggested a role for the NuRD complex and histone-deacetylation in AC invasion. Additional chromatin modifiers involved, such as components of the MEC complex, remain to be identified. It will be of interest to investigate how *egl-43* and *nhr-67* interact with the known (i.e., HDA-1 and MEP-1) and the yet to be identified epigenetic regulators of AC invasion. A potential function of *egl-43* in epigenetic gene regulation is suggested by the interactions of human EVI1 with different chromatin modifier complexes (Bard-Chapeau et al., 2013). For example EVI1 recruits the corepressor CrBP to the SMAD3 promoter to repress TGFb signaling (Izutsu et al., 2001), interacts with components of the SWI/SNF to de-repress E2F1 expression and binds to the polycomb complex to inhibit PTEN signaling (Chi et al., 2003; Yoshimi et al., 2011). Isoform-specific interactions of EVI1 have been observed with components of the NuRD complex (Ivanochko et al., 2019), suggesting that context dependency may also affect the interaction with epigenetic regulators. Thus, an analysis of EGL-43 protein interaction partners may reveal epigenetic regulators that are critical for establishing the invasive AC fate.

### Can the Dichotomy Between Proliferation and Invasion Be Applied to Cancer Cells?

A similar dichotomy between proliferation and invasion has been observed in human cancer cells. The “go or grow” concept states that cells must choose one of three options; (1) to proliferate, (2) to migrate, or to (3) terminally differentiate (Hatzikirou et al., 2012). In this context, the colony-stimulating factor-1 receptor CSF1R and the non-receptor tyrosine kinase and Arg/Abl2 are important players in regulating the invasion-proliferation switch in cancer (Gil-Henn et al., 2013; Patsialou et al., 2015). This dichotomy also manifests in many cancer cells that undergo EMT-like changes, which coincide with their proliferation arrest (Kohrman and Matus, 2017). It has also been proposed that cancer cells, especially in human melanoma, switch back and forth between an epithelial, proliferative and a mesenchymal, invasive state (Hoek et al., 2008) However, the direct observation of invading melanoma cells in real time has again challenged this model (Haass et al., 2014). In *C. elegans*, AC-specific expression of the p21 homolog CKI-1 restored the invasive fate even when the AC was induced to proliferate, suggesting a plasticity between the proliferative and invasive states. Similar to the situation in *C. elegans*, a switch between invasion and proliferation has been proposed for breast cancer based on the finding that loss of G1 phase inhibitor p21 (CKI) or overexpression of cyclin E lead to suppression of metastasis (Qian et al., 2013). Furthermore, a link between G1 cell cycle and invadopodia formation during breast carcinoma invasion has recently been reported (Bayarmagnai et al., 2019). Though, the fact that invadopodia precursors can be assembled throughout the cell cycle leaves a more nuanced picture of the proliferation-invasion switch (Bayarmagnai et al., 2019).

## Concluding Remarks

The *C. elegans* AC is an excellent model to investigate the various aspects underlying the complex process of cell invasion using an integrated approach by simultaneously examining: (1) cell fate acquisition, (2) establishment and maintenance of cell cycle arrest, (3) epigenetic and transcription factor networks that induce a pro-invasive gene expression pattern, (4) generation of extracellular cues that guide invading cells, (5) formation of invasive protrusions and finally (6) BM breaching. Since the AC does not migrate through the BM after breaching, it allows to separate cell invasion from later events occurring during cellular movements, thereby disentangling the different signaling pathways involved. Moreover, many transcription factors act in a context-dependent manner, underlining the importance of studying cell invasion in a physiological context. A remaining challenge is to understand the connections between the different layers controlling cell invasion, for example the link between G1 cell cycle arrest and the specification of the invasive fate. Many regulators of AC invasion are conserved and are associated with oncogenic processes in human cancer. Therefore, the unique AC of *C. elegans* could play a prominent role in solving the question of the proliferation-invasion interplay.

## Author Contributions

EL wrote the first draft of the manuscript. All authors contributed to manuscript revision, read and approved the submitted version.

## Conflict of Interest

The authors declare that the research was conducted in the absence of any commercial or financial relationships that could be construed as a potential conflict of interest.
